# Influence of Age and Cardiovascular Risk Factors in Vestibular Neuritis: Retrospective Cohort Study

**DOI:** 10.3390/jcm12206544

**Published:** 2023-10-16

**Authors:** Guillermo Salib Coronel-Touma, Chiara Monopoli-Roca, Cristina Nicole Almeida-Ayerve, Susana Marcos-Alonso, Diana Gómez de la Torre-Morales, José Serradilla-López, Santiago Santa Cruz-Ruiz, Ángel Batuecas-Caletrío, Hortensia Sánchez-Gómez

**Affiliations:** 1 Department of Otorhinolaryngology—Head & Neck Surgery, Hospital Universitario de Salamanca, 37007 Salamanca, Spain; cmonopoli@saludcastillayleon.es (C.M.-R.); cnalmeida@saludcastillayleon.es (C.N.A.-A.); smarcosal@saludcastillayleon.es (S.M.-A.); jmserradilla@saludcastillayleon.es (J.S.-L.); ssantacruz@saludcastillayleon.es (S.S.C.-R.); abatuecas@saludcastillayleon.es (Á.B.-C.); hortensiasanchez@saludcastillayleon.es (H.S.-G.); 2 Department of Neurology, Hospital Universitario de Salamanca, 37007 Salamanca, Spain; dsgomez@saludcastillayleon.es; 3 Biomedical Research Institute of Salamanca (IBSAL), Faculty of Medicine, The University of Salamanca, 37007 Salamanca, Spain

**Keywords:** vestibular neuritis, cardiovascular risk factors, vestibular-ocular reflex, video head impulse test

## Abstract

To analyze the influence of age and cardiovascular risk factors (CVRFs) in the evolution of vestibular neuritis (VN). Methods: Retrospective cohort study. VN-diagnosed patients were included and divided into two groups: those with and without CVRFs. We analyzed the mean vestibular-ocular reflex (VOR) gain, measured through the video head impulse test (vHIT) at the diagnosis and one-year follow-up. We conducted a factorial analysis of variance (ANOVA) to evaluate the effect of age, sex, and CVRFs in the mean VOR gain. Results: Sixty-three VN-diagnosed patients were included. There were no statistically significant differences in the mean VOR gain between both groups. However, in the subgroup analysis, there were statistically significant differences when comparing the mean VOR gain at the one-year follow-up between the group over 55 years of age 0.77 ± 0.20 and the group under 55 years 0.87 ± 0.15 (*p* = 0.036). Additionally, the factorial ANOVA demonstrated a significant main effect of age group on the mean VOR gain at the one-year follow-up (*p* = 0.018), and it also found a significant interaction between the factors of gender, age group, HTN (*p* = 0.043). Conclusions: CVRFs do not independently affect the mean VOR gain in VN patients’ follow-ups. However, age significantly impacts VOR gain in VN and could be modulated by gender and hypertension.

## 1. Introduction

Vestibular neuritis (VN) is an acute peripheral vestibular syndrome, also known as acute unilateral vestibulopathy. It is characterized by an acute unilateral loss of peripheral vestibular function in the absence of acute neurological or auditory signs and symptoms [1].

The diagnostic criteria were defined in 2022 by the committee for the classification of vestibular disorders of the Bárány Society [1]. Among peripheral vestibular disorders, VN is the third most common cause, following benign paroxysmal positional vertigo (BPPV) and Meniere’s disease, with an annual incidence ranging from 3.5 to 15.5 per 100,000 inhabitants [2]. It can occur between the ages of 30 and 60, with a higher prevalence between 40 and 50 years of age. There are no significant differences between men and women [3].

Viral etiology is the most likely cause but remains unproven to date [4,5,6,7]. There is histopathological evidence of degeneration and inflammation of the vestibular nerve [8], as well as the detection of herpes simplex virus type 1 [9,10]. However, similar signs and symptoms can be produced by an infarction in the anterior vestibular artery, which is more vulnerable than the inferior vestibular artery [11].

The prevalence of cardiovascular risk factors (CVRFs) associated with VN has not been sufficiently documented, and depending on the series, a higher or lower prevalence of CVRFs associated with VN has been reported [12,13].

The video head impulse test (vHIT) is a useful tool in the diagnosis of vestibular disorders: it assesses the vestibular-ocular reflex (VOR) by isolating the vestibular component. Therefore, it is valuable within the differential diagnosis of an acute vestibular syndrome, where an abnormal vHIT would rule out the possibility of encountering an acute unilateral vestibulopathy of central origin [14]. Furthermore, vHIT is commonly used in the follow-up of VN patients to objectively evaluate the restitution or readjustment of vestibular function [15,16].

Our study aims to assess the influence of age and CVRFs in the evolution of patients with VN through measurement of the mean VOR gain at diagnosis and one-year follow-up.

## 2. Materials and Methods

### 2.1. Study Design and Data Collection

A retrospective cohort study was conducted between January 2020 and December 2022 at our institution. Data gathering was conducted using clinical records from the otoneurology unit. VN-diagnosed patients were included, following the Bárány Society criteria. All of the VN-diagnosed patients included had a vHIT at the time of diagnosis and one-year follow-up to assess the VOR gain. The VOR was deemed normal if it was above 0.80, while below was considered pathological.

### 2.2. Selection Criteria

The inclusion criteria were as follows: (1) acute or subacute episode of moderate to severe vertigo lasting at least 24 h; (2) spontaneous nystagmus with peripheral characteristics (unidirectional, horizontal-rotatory, intensity decreasing with gaze fixation and increasing with gaze); (3) positive head impulse test in the direction of the fast phase of spontaneous nystagmus; (4) no evidence of acute neurological or audiological symptoms (tinnitus, hearing loss, or sensation of ear fullness); (5) patients over 18 years of age with and without CVRFs (hypertension, diabetes mellitus, and dyslipidemia); (6) vHIT performed at the time of diagnosis (during the acute episode or within one week after symptom onset) and at one-year follow-up; (7) pure-tone audiometry without evidence of hearing fluctuation or sudden hearing loss criteria; (8) nuclear magnetic resonance imaging without pathology in the posterior cranial fossa. Patients were excluded based on the following exclusion criteria: (1) patients with acute vestibular syndrome who do not meet the diagnostic criteria for vestibular neuritis according to the Bárány Society; (2) absence of vHIT at the time of diagnosis or at one-year follow-up; (3) absence of nuclear magnetic resonance imaging ruling out pathology in the posterior cranial fossa.

### 2.3. Cohort Groups, Subgroups, and Statistical Methods

First, in order to compare and analyze demographic (age, sex, and pathologic ear) and clinical (hypertension, diabetes, dyslipidemia, and the VOR gain at the diagnosis and one-year follow-up) features, sixty-three VN patients were divided into two groups: those with and without CVRFs. Then, we performed a subgroup analysis based on each CVRF and an age subgroup based on patients who were older and younger than 55 years old.

The statistical analysis was performed using IBM^®^ SPSS^®^ Statistics Version 25. Demographic and clinical features were described using descriptive statistics. Number and percentage were used for qualitative data. For normal distribution data, mean and standard deviation (SD) were used for quantitative data, while median and range were used for non-normal distribution data.

The qualitative variables under study were sex, pathological-side ear, CVRFs, hypertension (HTN), type II diabetes mellitus (DM), dyslipidemia, and age group (older and younger than 55 years old). The quantitative variables included age and the mean gain of VOR on the pathological-side at diagnosis and one-year follow-up.

Between groups and subgroups, qualitative variable analysis was conducted using the chi-squared test; qualitative and quantitative variables were analyzed using the Student’s t-test to compare the mean difference of the quantitative variables.

Finally, after analyzing the normal distribution of the quantitative variable under study (mean VOR gain at diagnosis and one-year follow-up on the pathological side) using the Kolmogorov–Smirnov normality test (*p* > 0.05), homoscedasticity using the Levene’s Test (*p* > 0.05), and ensuring independence, a factorial ANOVA was used to analyze the effect on the mean VOR gain at diagnosis and one-year follow-up on the pathological side, considering the CVRFs, gender, and age. Within the factorial ANOVA, multiple comparisons were conducted and adjusted for multiple comparisons using the Bonferroni post-hoc analysis. All tests achieved statistical significance with a *p*-value below 0.05.

## 3. Results

### 3.1. General Demographics and Clinical Characteristics

After an exhaustive review of clinical records, we found 102 VN-diagnosed patients; however, only 63 patients met the selection criteria and were included for the analysis. Mainly, 39 patients were excluded due to the absence of vHIT results at diagnosis or one-year follow-up, as well as the lack of magnetic resonance imaging of the posterior cranial fossa.

The mean age was 60 ± 15 years old, of which 52.4% were male and 47.6% were female. According to the laterality of the unilateral vestibular deficit, the pathological ear was left in 55.6% of the cases, while for the right ear, it was 44.4%. In our study, patients with CVRFs were predominant, accounting for 63.5%, of whom at least one of them under study was present. Out of the sixty-three VN patients, 47.6% had hypertension, 44.4% had dyslipidemia, and 11.1% had type 2 diabetes. Furthermore, the mean VOR gain of the pathological-side ear was 0.46 ± 0.15 at diagnosis, and 0.81 ± 0.18 at the one-year follow-up. Finally, at the end of the follow-up, 77.8% of the cases had a normal VOR gain, while 22.2% remained pathological (Table 1).

### 3.2. Comparison between Groups

A total of sixty-three VN-diagnosed patients were divided into two groups: those with and without CVRFs. (Table 2). Forty patients were included in the CVRFs group, whereas twenty-three patients were included in the control group.

The mean age was higher in the CVRFs group with 65 years ± 12 versus 50 years ± 15 in the control group (*p* < 0.001). Sex distribution was similar for both groups, with a slight predominance of males (52.5%) over females (47.5%) (*p* = 0.980). For both groups, more than 50% of the pathological side was the left side (*p* = 0.907).

Moreover, there were no statistically significant differences between both groups when comparing the mean VOR gain in the pathological-side ear at diagnosis and the one-year follow-up. At diagnosis, the mean VOR gain was similar for both groups: 0.47 ± 0.16 for the CVRFs group and 0.45 ± 0.15 for control group (*p* = 0.752). In addition, at the one-year follow-up, the mean VOR gain for the CVRFs group was 0.82 ± 0.18 and 0.81 ± 0.19 for control group (*p* = 0.952). However, the vHIT remains pathological in 25% of cases for the CVRFs group and 17.4% for the control group (*p* = 0.484).

### 3.3. Subgroup Analysis

Out of 40 VN patients with CVRFs, 47% presented hypertension, followed by 44% of patients with dyslipidemia, and finally 11% with type 2 diabetes.

There were no statistically significant differences for qualitative variables (sex, pathological-side ear, and result of vHIT at the end of the follow-up). Nevertheless, we found statistically significant differences between the mean age of VN patients with and without hypertension at 67 ± 13 versus 54 ± 16 years of age (*p* < 0.001), and dyslipidemia at 65 ± 11 versus 56 ± 17 years of age (*p* = 0.027), but not for type 2 diabetes at 69 ± 14 versus 59 ± 15 years of age (*p* = 0.102).

In addition, no statistically significant differences were found between the mean VOR gain of the pathological side of VN patients with and without hypertension, dyslipidemia, and type 2 diabetes at diagnosis and the one-year follow-up.

Furthermore, in our study, we subdivided the sixty-three VN patients based on the age into two groups: those over and under 55 years old. Thirty-eight patients were older than 55 years versus twenty-five patients who were younger that 55. The CVRFs were predominant in the group over 55: 30 versus 10 patients (*p* = 0.002). Moreover, there were more patients with hypertension in the over 55 group: 24 versus 6 patients (*p* = 0.002).

**Figure 1:**
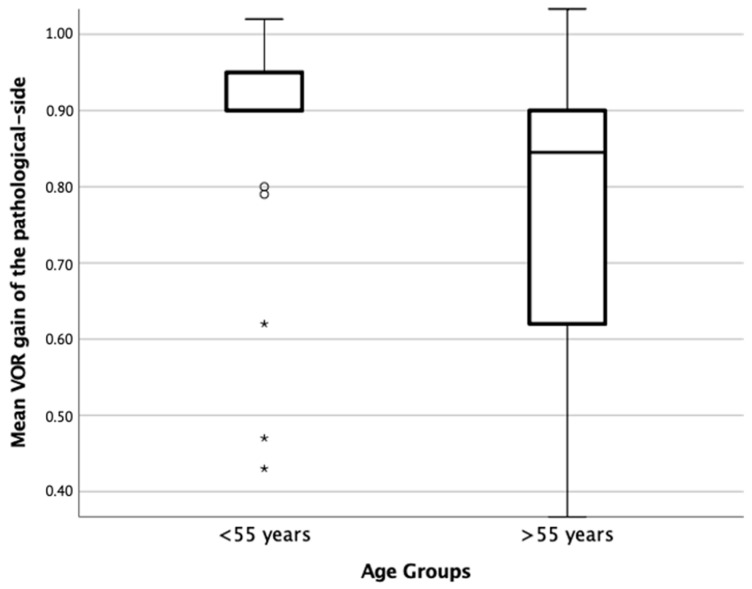
Mean VOR gain at the one-year follow-up between age groups. Circles represent the outliers and asterisks represent the extreme values. Both of these indicate patients with a VOR gain below 0.80, which is considered pathological.

There were no statistically significant differences when comparing the mean VOR gain of the pathological side at diagnosis between both groups: 0.46 ± 0.16 versus 0.47 ± 0.15 (*p* = 0.799); however, at the one-year follow-up, statistically significant differences were found when comparing the mean VOR gain of the pathological side between the group over 55 years old versus the group under 55 years old: 0.77 ± 0.20 versus 0.87 ± 0.15 (*p* = 0.046) (Figure 1).

Finally, we conducted a factorial ANOVA and multiple comparisons using Bonferroni post-hoc analysis to evaluate how various factors might influence the mean VOR gain of the pathological side at diagnosis and at the one-year follow-up. The model of the factorial ANOVA included age (over and under 55 years old), sex, HTN, dyslipidemia, and diabetes.

At diagnosis, the factors included in the model did not have a statistically significant main effect or interaction between them on the mean VOR gain. At the one-year follow-up, the factorial ANOVA showed that age had a significant effect on the mean VOR gain of the pathological side. The group under 55 years old had a higher recovery compared to the group over 55 years old (0.90 ± 0.043 versus 0.77 ± 0.031), (*p* = 0.018).

Sex, HTN, dyslipidemia, and diabetes did not have a statistically significant main effect on the mean VOR gain. However, there was a significant interaction between sex, age group, and HTN (*p* = 0.043).

**Figure 2:**
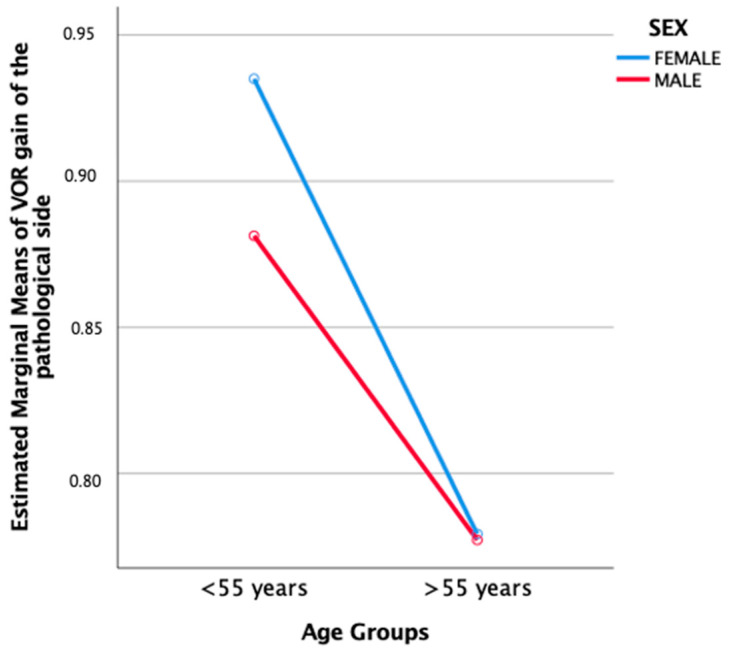
Estimated marginal means of VOR gain at the one-year follow-up demonstrated interaction between age groups and sex.

Multiple comparisons using Bonferroni post-hoc analysis indicated that between sex and age group (Figure 2), the group of females under 55 years old had a statistically significant higher recovery compared to the group of females over 55 years old (0.93 ± 0.064 versus 0.77 ± 0.040), (*p* = 0.043). Although the group of males under 55 years had a higher recovery compared to the group of males over 55 years old, it was not statistically significant (0.88 ± 0.058 versus 0.77 ± 0.048), (*p* = 0.171).

**Figure 3:**
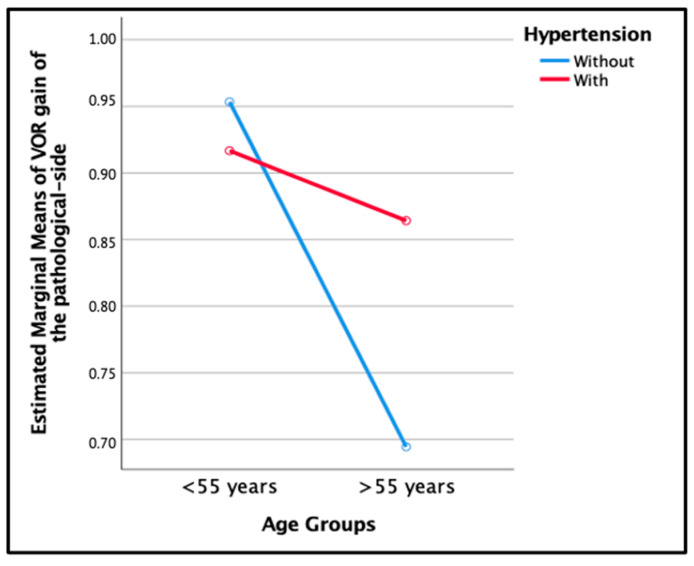
Estimated marginal means of VOR gain at the one-year follow-up demostrated interaction between age groups and hypertension in females.

Contrary to what was previously described, we observed that in the group of females over 55 years old with hypertension, there was a significantly higher recovery compared to the group of females over 55 years without hypertension (0.86 ± 0.052 versus 0.69 ± 0.060), (*p* = 0.037). In addition to this, in both groups of females under 55 years old with and without hypertension, the mean VOR gain was similar (0.91 ± 0.10 versus 0.95 ± 0.074), (*p* = 0.774), (Figure 3).

**Figure 4:**
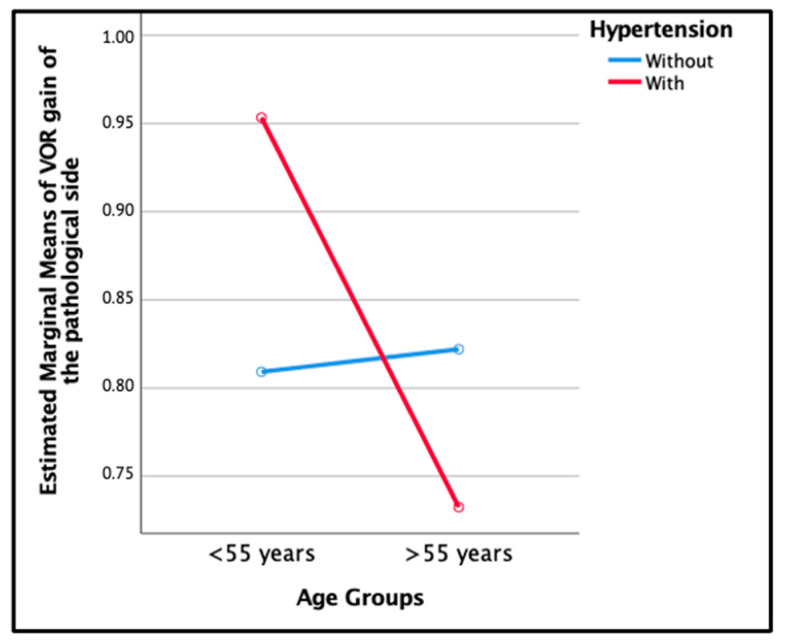
Estimated marginal means of VOR gain at the one-year follow-up demostrated interaction between age groups and hypertension in males.

Although it was not statistically significant, the group of males over 55 years old with hypertension had a lower mean VOR gain compared to the group without hypertension (0.73 ± 0.052 versus 0.82 ± 0.081), (*p* = 0.354), (Figure 4).

## 4. Discussion

In our study, we postulate that the evolution of VN patients could or could not be influenced by age and/or CVRFs. Our results suggest that ¨age¨, as an independent factor, and the interaction of ¨age, sex, and hypertension¨ had a significant effect on the mean VOR gain at the one-year follow-up in patients with VN. However, in our sample, CVRFs do not appear to influence the evolution of the VOR in patients with VN.

The majority of the VN patients included in our sample presented at least one of the studied CVRFs. Hypertension was the CVRF more commonly observed, followed by dyslipidemia, and type 2 diabetes mellitus. Contrarily, Pâris et al. [12] concluded that superior vestibular neuritis patients did not present more prevalence of CVRFs in comparison with the French general population, even with significantly lowered cholesterol levels. Nevertheless, Oron et al. [17] concluded that there may be an interrelation between CVRFs and VN based on the hypothesis of affected vestibular nerve’s microvascularization in VN. They also found the prevalence of more CVRFs in VN-hospitalized patients in comparison with the Israeli general population.

Even though the majority of VN patients presented CVRFs, in our study, no statistically significant differences were found between the CVRFs group and the control group when comparing the mean VOR gain on the pathological side, neither at diagnosis nor at one-year follow-up, which could indicate that CVRFs, on their own, do not influence the evolution of the VOR. According to Chung et al. [18], the CVRFs had a limited value in predicting the clinical evolution of patients with VN as no significant correlations were found between arterial stiffness, metabolic syndrome scores, and clinical parameters of vestibular neuritis, such as dizziness disability scores and vestibular function tests, including caloric tests and vHIT.

In addition, we observed that the VN with CVRFs group had a significantly higher mean age than those without CVRFs. This difference could suggest that older patients with CVRFs are at greater risk of developing VN. Additionally, it was noted that the mean age of VN patients varied depending on the type of CVRF present. Patients with hypertension and dyslipidemia had a significantly higher mean age than those without these conditions. Furthermore, Chung et al. [18] investigated the clinical significance of CVRFs, including arterial stiffness and metabolic syndrome scores, in the development of VN and found that blood pressure, brachial-ankle pulse velocity, and metabolic syndrome scores were higher in the VN group compared to the control group, concluding that these factors are associated with the development of VN.

Yan et al. [19] investigated the clinical characteristics of VN patients among different age groups and found that the prevalence of hypertension and diabetes mellitus showed a significantly increasing trend from young adults to older individuals. Caloric response was significantly worse in the older age group compared to the other groups. Abnormal rates in the vHIT test, vestibular evoked myogenic potential, and vestibular autorotation test did not differ significantly among the different age groups.

Could age influence or have an effect on the mean VOR gain of the pathological-side at one-year follow-up? When subdividing the sample by age, it was observed that CVRFs, particularly the presence hypertension and dyslipidemia, were significantly more common in the 55 years older group of patients compared to the group under 55 years old. These findings suggest that age could be a determining factor in the occurrence of CVRFs in patients with VN, and consequently, the relationship between age and the prevalence of CVRFs could have implications in the evolution of VN patients in different age groups. According to Yan et al. [19], abnormal rates in the horizontal plane of the vHIT varied from 77.8% (adolescents) to 91.3% (young adults), and 94.7% (middle-aged adults) up to 100% (older adults), showing an increasing trend with age, though not statistically significant.

In our study, although there were no statistically significant differences in the mean VOR gain at diagnosis between the 55 years older group in comparison with the under 55 years old group, at the one-year follow-up, significant differences were observed in the mean VOR gain of the pathological-side. Patients under 55 years of age showed a significantly higher mean VOR gain compared to the 55 years older group. This suggests that age could influence the recovery of vestibular function in patients with VN, with a better prognosis in younger patients. On the contrary, it is important to consider what was described by Jay et al. [20], who, in their study, found a significant correlation between age and VOR gain in individuals over 58 years old. Furthermore, they confirmed previous findings that link age to the saccade profile in vHIT, suggesting that small saccades may indicate age-related vestibular impairment that is not reflected in obvious changes in VOR gain.

The results of the factorial ANOVA in our study demonstrated valuable insights into the factors influencing the VOR gain of a patient with VN, at diagnosis and at the one year follow-up.

At diagnosis, none of the factors we considered (age, sex, hypertension, dyslipidemia, and diabetes) had a significant effect on or interaction with the VOR gain.

However, at the one-year follow-up, age emerged as a significant factor. Specifically, individuals under 55 years old showed a higher recovery compared to those over 55 years old. This indicates that younger individuals may have a better prognosis in terms of VOR gain recovery.

While sex, hypertension, dyslipidemia, and diabetes did not, individually or by association, have a significant effect on the mean VOR gain, there was a significant interaction observed between sex, age group, and hypertension. Our post-hoc analysis further revealed that among females under 55 years old, there was a significantly higher recovery compared to females over 55 years old. However, this age-related difference was not observed in males.

Contrary to expectations, it was observed that females over 55 years old with hypertension showed a significantly higher recovery rate when compared to those without hypertension, and males over 55 years old with hypertension had a lower mean VOR gain compared to those without hypertension. These results suggest that, in our study, HTN did not have a significant effect on the mean VOR gain despite sex and age.

### Limitations

The risk of bias associated with a retrospective study conducted in a single hospital center. The small sample size results in heterogeneous groups for analysis and could impact the results. The absence of other CVRFs that could be subject to analysis and influence the evolution of patients with VN, such as ischemic heart disease, atrial fibrillation, metabolic syndrome, habits such as smoking or alcohol consumption, etc. Subjective dizziness is unrelated to the gain of VOR; even if the gain of VOR is normal or pathological, this is an important limitation that has to be taken into account for further studies. Additionally, it might be of greater interest to analyze the difference in VOR gain between the healthy side and the pathological side of unilateral vestibular deficit. In contrast, our study employed a factorial ANOVA with significant results, and the post-hoc comparisons specify where the statistically significant differences are found, such as factors that have a main effect and a significant interaction with the mean VOR gain of the pathological side at the one-year follow-up in patients with VN.

Finally, for future research, we recommend conducting prospective studies to investigate the potential role of vascular disease as a possible etiological factor in VN, along with exploring the association between age, sex, and CVRFs in the evolution of patients with VN.

## 5. Conclusions

According to our study, patients with VN and CVRFs are older on average than patients without CVRFs; however, there are no statistically significant differences in the mean VOR gain at diagnosis or follow-up between the groups with and without CVRFs.

Factors such as age and CVRFs did not significantly impact the VOR gain at diagnosis. However, at the one-year follow-up, age became a significant factor, with patients under 55 showing higher recovery rates compared to patients over 55 years old. An interaction between sex, age group, and HTN was observed. Females under 55 years had a higher recovery rate compared to those over 55 years old, a significant difference not seen in males. Additionally, hypertensive females over 55 years showed higher recovery than those without HTN. This suggests that in our study, the presence of HTN did not significantly impact the VOR gain.

## Figures and Tables

**Table 1:** General demographics and clinical characteristics.

	Vestibular Neuritis
	N = 63 (%)
Mean age (years)	60 ± 15
Sex	
Male	33 (52%)
Female	30 (48%)
Age Subgroup	
<55 years	24 (38%)
>55 years	39 (62%)
Pathological-side ear	
Left ear	35 (56%)
Right ear	28 (44.4%)
^1^ CVRFs	
Hypertension	30 (48%)
Dyslipidemia	28 (44%)
Type 2 Diabetes	7 (11.1%)
Diagnosis mean ^2^ VOR gain	0.46 ± 0.15
One-year follow-up mean VOR gain	0.81 ± 0.18
^3^ vHIT result one-year follow-up	
Normal	49 (78%)
Pathological	14 (22%)

^1^ CVRFs—cardiovascular risk factors; ^2^ VOR—vestibular-ocular reflex; ^3^ vHIT—video head impulse test.

**Table 2:** Comparison between CVRFs and the control group.

	^1^ CVRFs Group	Control Group	*p*
	N = 40 (%)	N = 23 (%)	
Mean age (years)	65 ± 12	50 ± 15	<0.001
Sex			
Male	21 (52.5%)	12 (52.2%)	
Female	19 (47.5%)	11 (47.8%)	
Pathological-side ear			
Left ear	22 (55%)	13 (56.5%)	
Right ear	18 (45%)	10 (43.5%)	
Diagnosis mean ^2^ VOR gain	0.47 ± 0.16	0.45 ± 0.15	
One-year follow-up mean VOR gain	0.82 ± 0.18	0.81 ± 0.19	
^3^ vHIT result one-year follow-up			
Normal	30 (75%)	19 (82.6%)	
Pathological	10 (25%)	4 (17.4%)	

^1^ CVRFs—cardiovascular risk factors; ^2^ VOR—vestibular-ocular reflex; ^3^ vHIT—video head impulse test.

## Data Availability

Data are unavailable due to privacy or ethical restrictions.
